# Effects of Lightning on Rhizosphere Soil Properties, Bacterial Communities, and Active Components of *Camellia sinensis* var. *assamica*

**DOI:** 10.3389/fmicb.2022.911226

**Published:** 2022-05-23

**Authors:** Yaping Chen, Qiang Li, Wendou Wu, Xiaohui Liu, Jie Cheng, Xiujuan Deng, Xiaobo Cai, Wenxia Yuan, Jin Xie, Shihao Zhang, Baijuan Wang

**Affiliations:** ^1^College of Tea Science, Yunnan Agricultural University, Kunming, China; ^2^College of Plant Protection, Yunnan Agricultural University, Kunming, China; ^3^Yunnan Organic Tea Industry Intelligent Engineering Research Center, Kunming, China; ^4^College of Food and Biological Engineering, Chengdu University, Chengdu, China; ^5^College of Big Data, Yunnan Agricultural University, Kunming, China; ^6^Key Laboratory of Intelligent Organic Tea Garden Construction in Universities of Yunnan Province, Kunming, China

**Keywords:** lightning, microecology, biological effect, interaction, Pu-erh tea

## Abstract

Lightning rods have been developed to prevent damage caused by lightning to organisms. However, the biological effect of the current transmitted into the soil through lightning rods is unknown. In this study, we analyzed the effects of lightning on soil properties, the microbial community, and the active components of Pu-erh tea (*Camellia sinensis* var. *assamica*) near lightning rods. The results showed that the contents of organic matter and available potassium, copper, and calcium in rhizosphere soil near the lightning rod were significantly higher than those in control soil (*P* < 0.05), while the contents of total potassium, phosphorus, iron, magnesium, and aluminum decreased. Lightning significantly increased the bacterial diversity of Pu-erh rhizosphere soil compared to control soil samples (*P* < 0.05). *Sphingomonas, Nitrospira*, and *Reyranella* were significantly enriched in soil samples near the lightning rod compared to soil samples far from the lightning rod. Clusters of Orthologous Groups (COG) and Kyoto Encyclopedia of Genes and Genomes (KEGG) analyses indicated that adenosine/AMP kinase, chitodextrinase, flavorubredoxin, nucleotide metabolism, and carbohydrate digestion and absorption were significantly enriched in the rhizosphere soil samples near the lightning rod compared to the control samples (*P* < 0.05). β diversity analysis indicated the grounding of the lightning rod contributed to the community differentiation of rhizosphere bacteria. Amino acids, polyphenols, and soluble sugar increased in Pu-erh tea near the lightning rod, while the contents of catechin and anthocyanin decreased in Pu-erh tea near the lightning rod compared with the control sample (*P* < 0.05). Significant correlations were found among microbial indicators, soil properties, and Pu 'er tea components. This study serves as the first report on the effects of lightning rods on soil properties, microecology, and plant metabolism, which promotes the understanding of the biological effects of lightning, and provides a reference for the rational use of lightning resources.

## Introduction

Lightning is a magnificent and somewhat daunting discharge phenomenon in nature. Lightning generally occurs in cumulonimbus clouds with strong convection. In the process of air convection, electric charge is generated in the clouds (Cooray, 2003). The distribution of charge in the cloud is complex, but generally speaking, the upper part of the cloud is dominated by positive charge, and the lower part is dominated by negative charge. Therefore, a potential difference is formed between the upper and lower parts of the cloud. When the potential difference reaches a certain degree, discharge will occur (Dwyer and Uman, 2014; Cooray, 2015). The average current of lightning is 30,000 amps, and the maximum current can reach 300,000 amps. The voltage of lightning is very high, at about 100 million to 1 billion volts. The power of a moderate thunderstorm can reach 10 million watts, which is equivalent to the output power of a small nuclear power plant (Heidler et al., 1999; Bazelyan and Raizer, 2000). When charged thunderstorms approach protrusions on the ground, intense discharge can occur.

Lightning is usually considered harmful, as it can cause animal and plant injuries and forest fires (Gomes, 2012). Human beings have devised various methods to deal with the adverse effects of lightning (Betz et al., 2009; Rakov and Rachidi, 2009), and lightning rods are considered to be one effective method for doing this (Moore et al., 2000; Zeng et al., 2016). When lightning occurs, strong current is guided into the soil through the lightning rod. Soil is considered to be a huge capacitor. The effect of electric current on soil properties and microorganisms within a limited distance (<10 m) of a lightning strike is currently unknown. We hypothesized that lightning can affect soil properties, microbial communities and ultimately plant metabolism in the rhizosphere of nearby plants to some extent through lightning rods. Lightning contains huge amounts of energy and is a natural energy source that can be used. However, human use of lightning resources is very limited. A limited number of studies have shown that lightning can affect the content of ground pollutants and mediate the horizontal transfer of soil microbial genes (Rakov and Rachidi, 2009; Kotnik, 2013; Gharaylou et al., 2020). In addition, there are 500 million tons of nitrogen fertilizer synthesized by thunderstorms in the world every year (Chameides et al., 1977; Tie et al., 2002).

Xishuangbanna in Yunnan Province is located on the southwestern border of China and has many Pu-erh tea gardens. Pu-erh tea is a world-famous tea with a range of health values and contains a variety of active substances, including catechins and tea polyphenols (Huang et al., 2019; Yang et al., 2019; Liu et al., 2021). Owing to its unique climatic and geographical conditions (Reeve and Toumi, 1999; Williams, 2005), Xishuangbanna has a high frequency of thunderstorms and is the center of strong thunderstorms in China, with an annual average of more than 110 thunderstorm days. In this study, three 20-meter-high lightning rods and lightning arresters were used to connect the lightning rods into the soil to detect the effects of lightning on the soil physical and chemical properties, rhizosphere microbial community, and Pu-erh tea metabolism. The research results will help to reveal the interaction between lightning and soil abiotic and biological factors, and will provide ideas for the safe use of natural lightning resources and the green improvement of tea quality.

## Materials and Methods

### Experimental Design and Sample Collection

We chose to conduct the trial in a Pu-erh tea garden in Xishuangbanna, Yunnan, covering an area of 1,000 × 2,000 m^2^. In this Pu-erh tea (*Camellia sinensis* var. *assamica*) garden with three 20-m high lightning rods and each has a flasher to connect the lightning rods to the soil, we counted the average number of thunderstorms per year as being 115. The distance between each lightning rod is about 50 m (E100°30′16″;N21°46′7″). The lightning rods were installed at a distance of 10 m from the tea seedlings. Three seedlings close to the three lightning rods were set as the lightning treatment group. At the time of the lightning strike test until year 6, we sampled the rhizosphere soil of Pu-erh tree near the lightning rod to analyze the soil's physicochemical properties and microbial communities. We collected rhizosphere soil by shaking the soil attached to the surface of plant roots, and further removed plant residual tissue by sterilized tweezers. The rhizosphere soil microbial communities were analyzed by 16S high-throughput sequencing. In addition, the leaves of the tea plants were collected to analyze the contents of active components, including water extracts, catechins, flavones, anthocyanins, soluble sugar, polyphenols, and amino acids. The rhizosphere soil and leaves of tea plants in tea plantations far from the lightning rods (>1,500 m) were collected and used as control samples, which can ignore the influence of lightning. All groupings contained three biological replicates.

### Soil Physicochemical Assays

The rhizosphere soil of Pu-erh tea near and far from the lightning rods was collected for soil physicochemical analysis. We determined the soil organic matter content using the Tyurin method (Kalembasa and Jenkinson, 1973). Total nitrogen was detected using the Kjeldahl method (Kirk, 1950). Ammonium nitrogen in soil was measured using indophenol blue colorimetry, while nitrate nitrogen was measured using phenol disulfonic acid colorimetry. We also measured the total phosphorus in the rhizosphere soil using molybdenum antimony ascorbic acid spectrophotography. Available phosphorus in rhizosphere soil was determined using the baking soda leaching-molybdenum antimony colorimetric method. Total potassium was determined by flame photometry. Available potassium was detected using ammonium acetate extraction-flame photometry. Soil Ca, Mg, Na, Al, Cu, Fe, Mn, and Zn were determined by inductively coupled plasma optical emission spectroscopy (Optima 2000 DV; PerkinElmer, USA), with yttrium as the internal standard.

### Pu-erh Tea Component Detection

The tea catechin content was determined using high-performance liquid chromatography (HPLC) according to a previous method (Li et al., 2012). Tea polyphenols were detected using a Tea polyphenols assay kit (ZC-A1206, ZCI BIO, Shanghai, China). A Plant soluble sugar assay kit (YX-W-C502, Sinobestbio, Shanghai, China) was used to detect soluble sugars in Pu-erh tea. We measured the amino acid, total flavonoid, and anthocyanin contents of Pu-erh tea using an Amino acid assay kit (YX-W-A904), a Plant flavonoid assay kit (YX-W-A506), and a Plant anthocyanin content assay kit (YX-W-A515), respectively, which were purchased from Sino Best Biological Technology Co., Ltd. (Shanghai, China). Hot water leachates of tea were measured according to a previously described method (Li et al., 2021).

### Soil DNA Extraction and 16S rRNA Sequencing

A total of six rhizosphere soil samples were collected and transported to the laboratory in an ice bag. We extracted the total genome DNA from soil samples using a soil DNA kit (D5625-02, OMEGA, CA, USA). Extracted DNA was monitored on 1% agarose gels. We amplified the 16S rRNA V3-V4 regions of these samples using the primers (341F: 5′-CCTACGGGAGGCAGCAG-3′; 806R: 5′-GGACTACNVGGGTWTCTAAT-3′) with barcodes. We then purified the PCR products with a Qiagen Gel Extraction Kit (Qiagen, Germany). Sequencing libraries were generated using a TruSeq® DNA PCR-Free Sample Preparation Kit (Illumina, San Diego, California, USA). We assessed the library quality using a Qubit@ 2.0 Fluorometer (Thermo Scientific, Waltham, MA, USA) and an Agilent Bioanalyzer 2100 system (Agilent, Beijing, China). The library was sequenced on an Illumina HiSeq platform, and 250-bp paired-end reads were generated. FLASH V1.2.7(Magoc and Salzberg, 2011) was used to merge the paired-end reads. QIIME V1.9.1 (Caporaso et al., 2010) was used to conduct quality control and to obtain high-quality clean tags. Chimera sequences were removed based on the reference Silva database (Quast et al., 2013).

### OTU Cluster and Species Annotation

Sequences with ≥97% similarity were assigned to the same operational taxonomy units (OTUs) using Uparse software v7.0.1001 (Edgar, 2013). We further annotated representative sequences for each OTU. The Silva Database (Quast et al., 2013) was used to annotate the taxonomic information of each representative sequence based on the Mothur algorithm. We further normalized OTU abundance information using a standard sequence number corresponding to the sample with the fewest sequences. We performed alpha diversity and beta diversity analyses based on the output-normalized data.

### Alpha and Beta Diversity Analyses

The complexity of species diversity for each sample was analyzed based on 6 indices: observed-species, Chao1, Shannon, Simpson, Good's coverage, and PD_whole_tree. QIIME v1.7.0 (Caporaso et al., 2010) was used to calculate all indices, which were further displayed with R software v2.15.3. We selected two indices to identify community richness, i.e., observed-species and Chao1 estimator (http://www.mothur.org/wiki/Chao). Community diversity was identified through three indices: the Shannon index (http://www.mothur.org/wiki/Shannon), Simpson index (http://www.mothur.org/wiki/Simpson), and PD whole-tree index. Sequencing depth was characterized by Good's coverage (http://www.mothur.org/wiki/Coverage). Differences between samples in terms of species complexity were evaluated by beta diversity. We conducted non-metric multidimensional scaling (NMDS) analysis using the vegan package of R software.

### Functional Prediction and Statistical Analysis

Phylogenetic Investigation of Communities by Reconstruction of Unobserved States (PICRUSt) was used to determine the function of rhizosphere bacteria (Douglas et al., 2018) based on the OTU table. We further analyzed the COG (Ashburner et al., 2000) and KEGG (Kanehisa and Goto, 2000) functions of rhizosphere bacteria. The obtained results were expressed as mean values ± standard deviation (SD). We used SPSS 19 statistical software to determine the variance of samples through two-way ANOVA and Duncan's test. Significant variation between samples was determined at the *P* < 0.05 level.

## Results

### Effects of Lightning on Soil Properties

**Figure 1:**
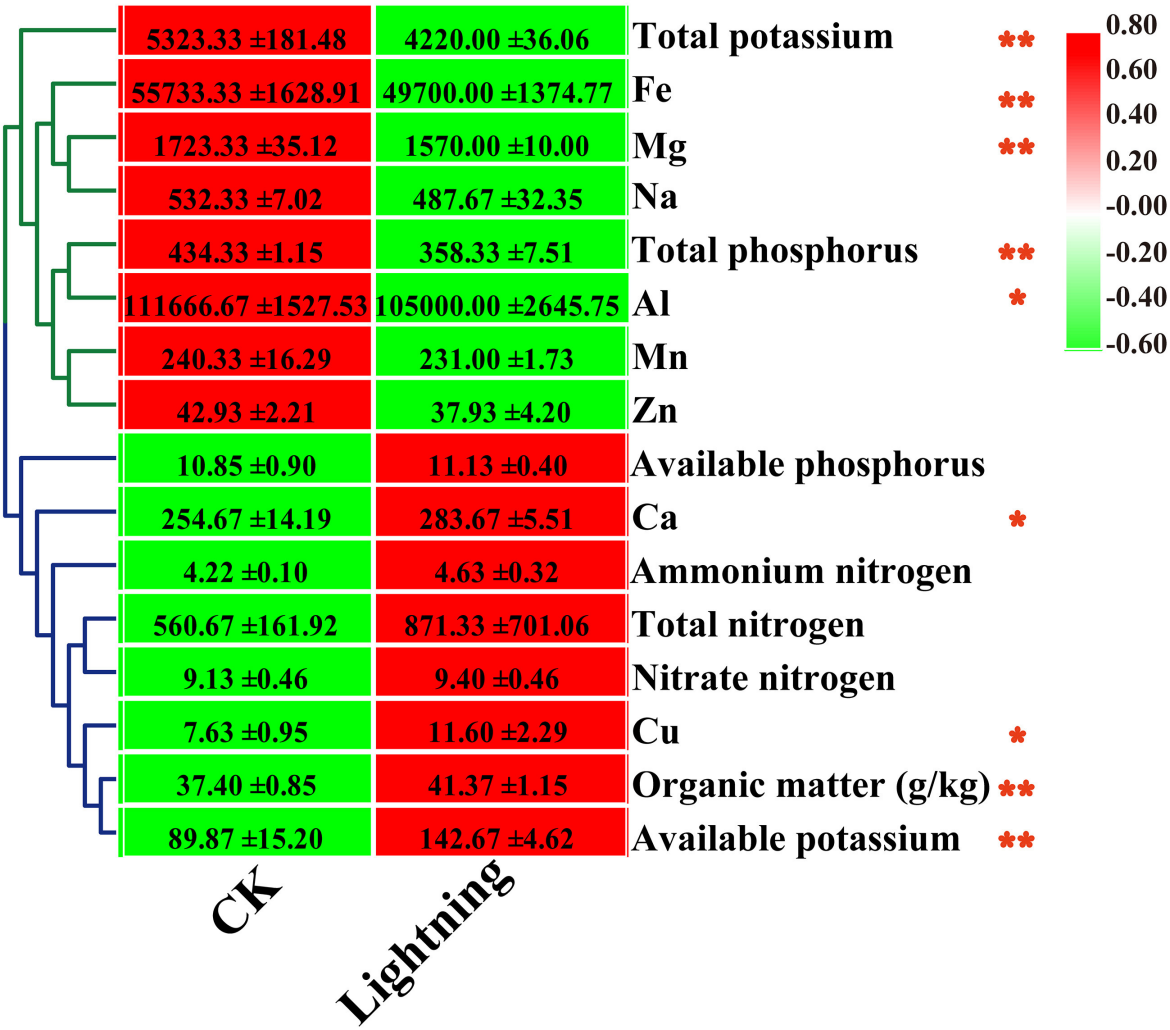
Heat map of rhizosphere soil properties of Pu-erh tea. The contents of different elements are expressed as mean values ± standard deviation (SD) (mg/kg). Lighting represents a sample 10 m from the lightning rod; CK indicates that the distance between the sample and the lightning rod is >1,500 m. *Indicates significant differences between different samples at the level of *P* < 0.05, while **Indicates significant differences between different samples at the level of *P* < 0.01.

We determined the effect of lightning on the chemical properties of the rhizosphere soil of nearby Pu-erh trees (Figure 1). The results showed that compared with the control, 8 of the 15 indexes increased to varying degrees in Pu-erh rhizosphere soil near the lightning rod, while 7 indexes decreased compared with the control soil. Statistical analysis showed that the contents of organic matter, available potassium, copper, and calcium in the rhizosphere soil near lightning rod were significantly higher than those in the control soil (*P* < 0.05). The contents of total potassium, total phosphorus, iron, magnesium, and aluminum in the rhizosphere soil near the lightning rod were significantly lower than those in the control soil (*P* < 0.05). The total potassium content of the Pu-erh tea rhizosphere soil near the lightning rod decreased, while the available potassium content increased compared with the control soil.

### Variation of Bacterial Alpha Diversity

**Figure 2:**
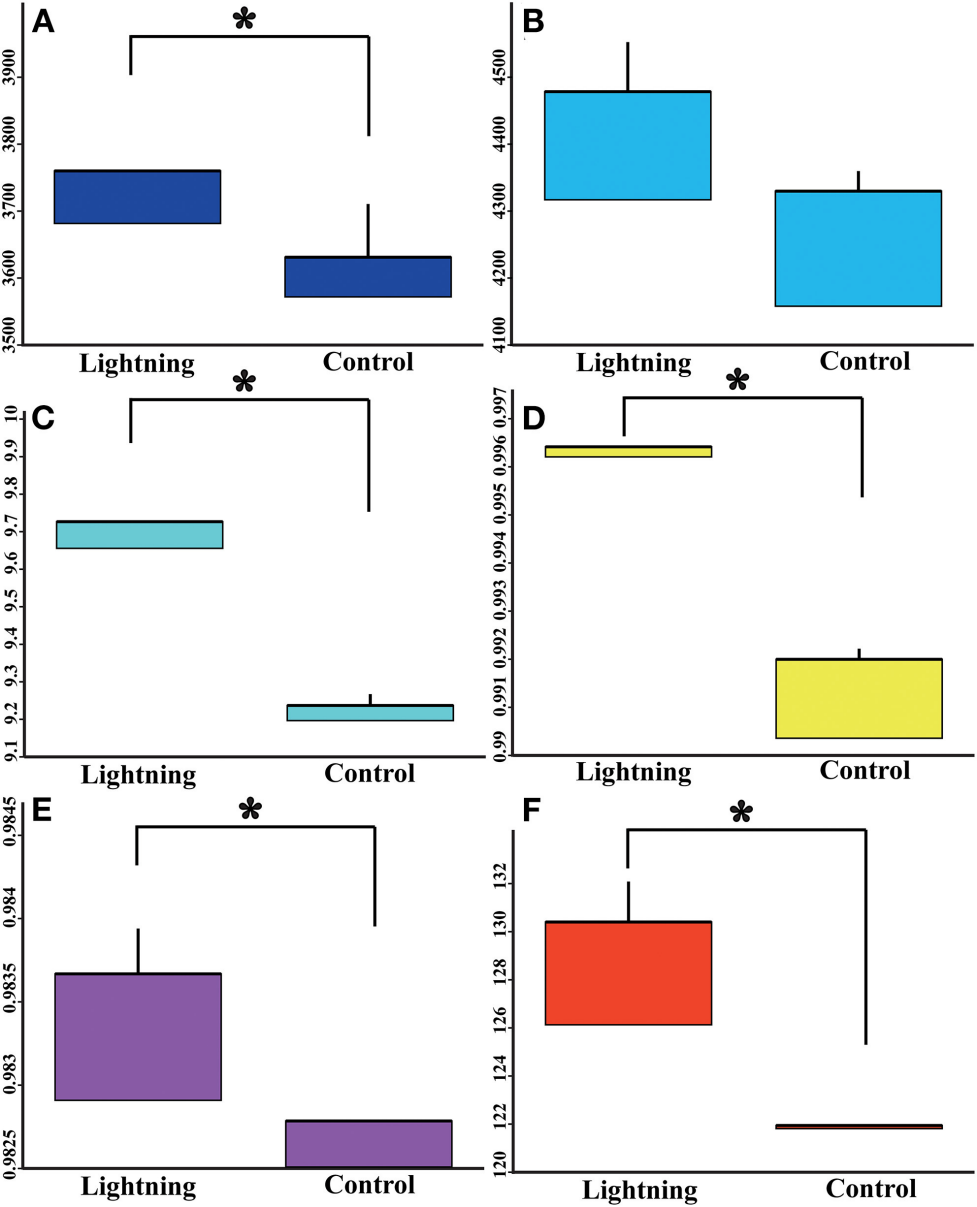
Box diagram of alpha diversity index of different samples. Lighting represents a sample 10 m from the lightning rod; Control indicates that the distance between the sample and the lightning rod is >1,500 m. *Indicates significant differences between different samples at the level of *P* < 0.05. **(A)** observed species index; **(B)** Chao 1 index; **(C)** Shannon index; **(D)** Simpson index; **(E)**, goods coverage; **(F)** PD whole tree.

The rarefaction curves of bacterial OTUs in different samples are shown in Supplementary Figure S1. With the increase in the number of sequencing reads, the number of observed species gradually increased, and the rarefaction curves gradually flattened after the sequencing reads exceeded 39,010, indicating that the sequencing reads were sufficient to reflect the overall community structure of the rhizosphere bacteria. We further analyzed the effects of lightning on the bacterial alpha diversity in the rhizosphere soil, including the community richness index, community diversity index, and sequencing depth index (Figure 2). In terms of community richness, lightning significantly increased the observed species index (*P* < 0.05), but had no significant effect on the Chao1 index. Lightning also significantly increased the community diversities, including the Shannon index, Simpson index, and PD whole-tree index (*P* < 0.05). In addition, the sequencing depth index, Good's coverage, also increased in the Pu-erh tree rhizosphere soil near the lightning rod compared with the control sample (*P* < 0.05).

### Taxonomic Analyses of Bacterial Communities

**Figure 3:**
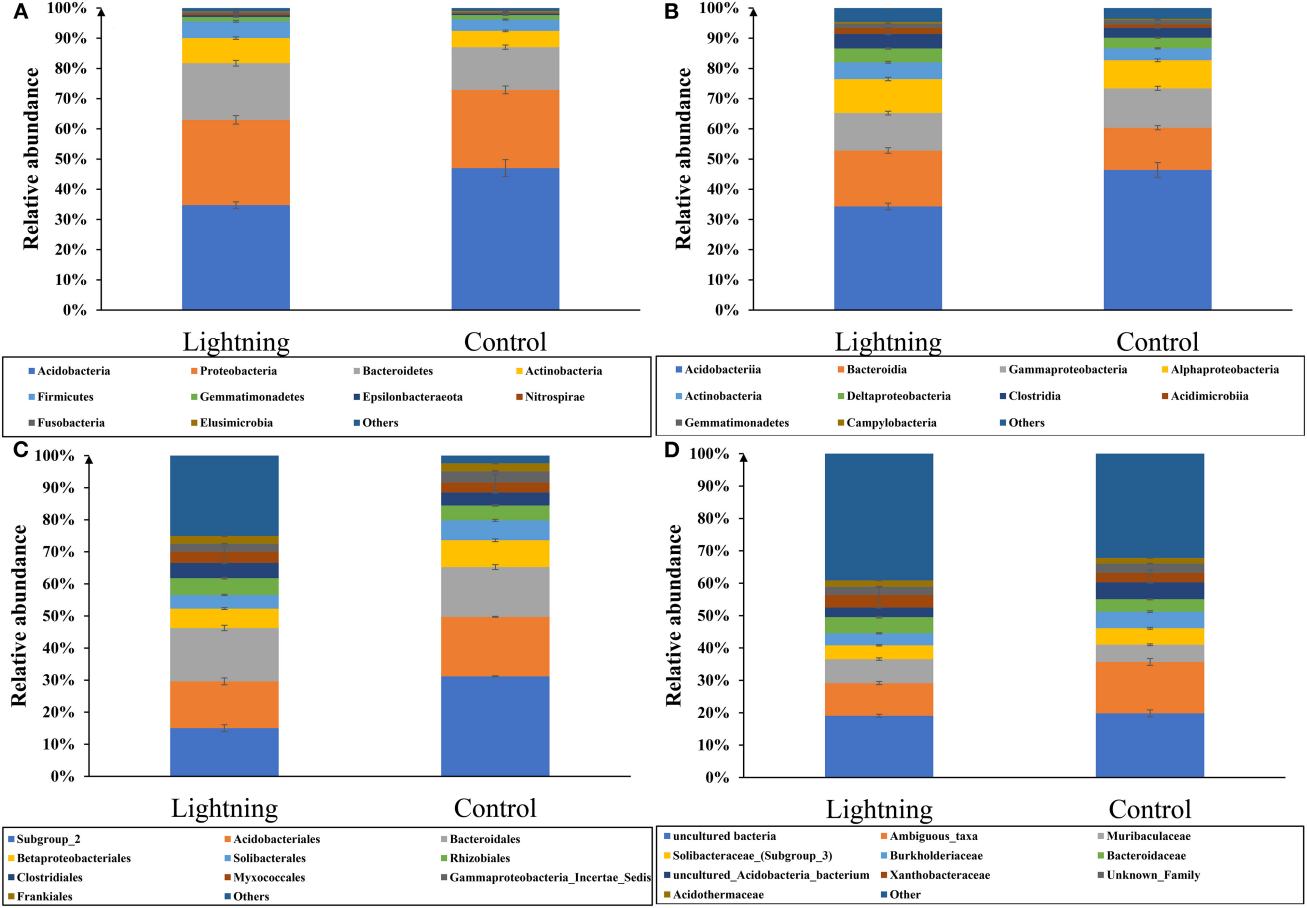
Relative abundance of taxa (top 10) at the phylum **(A)**, class **(B)**, order **(C)**, and family **(D)** level. Lighting represents a sample 10 m from the lightning rod; Control indicates that the distance between the sample and the lightning rod is >1,500 m.

We analyzed the difference in the taxonomic abundance between the rhizosphere soil sample near the lightning rod and the control sample (Figure 3A). The results showed that Acidobacteria was the most abundant phylum in all samples, accounting for 40.90% of rhizosphere bacteria, followed by Proteobacteria (average 27.04%), Bacteroidetes (average 16.45%), and Actinobacteria (average 6.87%). The CK sample contained the highest abundance of Acidobacteria (average 47.03%), which decreased significantly in the rhizosphere soil sample near the lightning rod (*P* < 0.05). The rhizosphere soil sample near the lightning rod had a higher abundance of Proteobacteria, Bacteroidetes, and Actinobacteria than the CK sample (*P* < 0.05).

Acidobacteriia, Bacteroidia, Gammaproteobacteria, and Alphaproteobacteria were the most abundant classes in all samples, accounting for 40.36, 16.26, 12.71, and 10.27% of rhizosphere bacteria, respectively (Figure 3B). Acidobacteriia decreased significantly in the soil sample near the lightning rod compared with the control sample (*P* < 0.05), whereas Bacteroidia and Alphaproteobacteria increased significantly in the soil sample near the lightning rod compared with the control sample (*P* < 0.05).

At the order level, Subgroup_2, Acidobacteriales, Bacteroidales, Betaproteobacteriales, and Solibacterales were the most abundant orders in all samples, accounting for 20.25, 14.88, 14.66, 6.44, and 4.67%, respectively, of rhizosphere bacteria (Figure 3C). The CK sample contained the highest abundance of Subgroup_2 (average 25.48%), which decreased significantly in the rhizosphere soil sample near the lightning rod (*P* < 0.05). The rhizosphere soil sample near the lightning rod had a higher abundance of Bacteroidales and Solibacterales than the CK sample (*P* < 0.05).

At the family level, uncultured bacteria, Ambiguous_taxa, Muribaculaceae, Solibacteraceae_(Subgroup_3), and Burkholderiaceae were the most abundant families in all samples (Figure 3D), which accounted for 19.46, 12.98, 6.34, 4.67, and 4.43%, respectively, of rhizosphere bacteria. The CK sample had the highest abundance of Ambiguous_taxa (average 15.87%), which decreased significantly in the rhizosphere soil sample near the lightning rod (*P* < 0.05). The rhizosphere soil sample near the lightning rod had a higher abundance of Muribaculaceae than the CK sample (*P* < 0.05).

**Figure 4:**
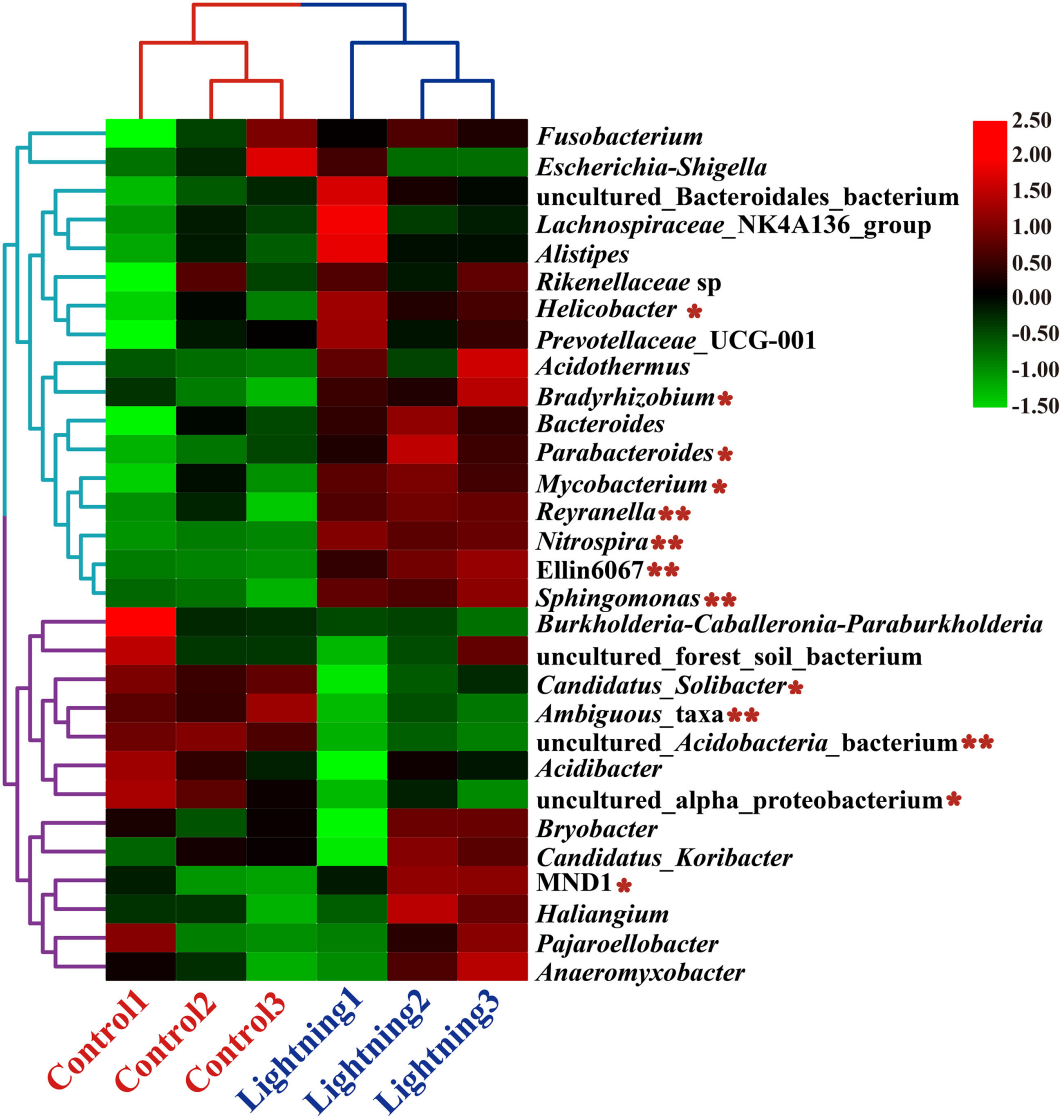
Heat maps of the 30 genera with the highest relative abundance in rhizosphere soils of Pu-erh tea. The color block in the heat map from green to red indicates that the abundance of the sample increases gradually. Lighting represents a sample 10 m from the lightning rod; Control indicates that the distance between the sample and the lightning rod is >1,500 m. *Indicates significant differences between different samples at the l0065vel of *P* < 0.05, while ** indicates significant differences between different samples at the level of *P* < 0.01.

Among the 30 genera with the highest abundance in all samples, the relative abundance of 17 genera increased and that of 13 decreased significantly in the rhizosphere soil samples near the lightning rod compared with the control samples (*P* < 0.05) (Figure 4). Genera with significant increases in the soil samples near the lightning rod included *Sphingomonas, Nitrospira, Reyranella, Mycobacterium, Parabacteroides, Bradyrhizobium*, and *Helicobacter*. By contrast, the relative abundance of MND1, uncultured_alpha_proteobacterium, uncultured_Acidobacteria_bacterium, Ambiguous_taxa, and *Candidatus_Solibacter* significantly decreased in the rhizosphere soil samples near the lightning rod compared with the control samples (*P* < 0.05).

### Functional Differentiation of Rhizosphere Bacteria

**Figure 5:**
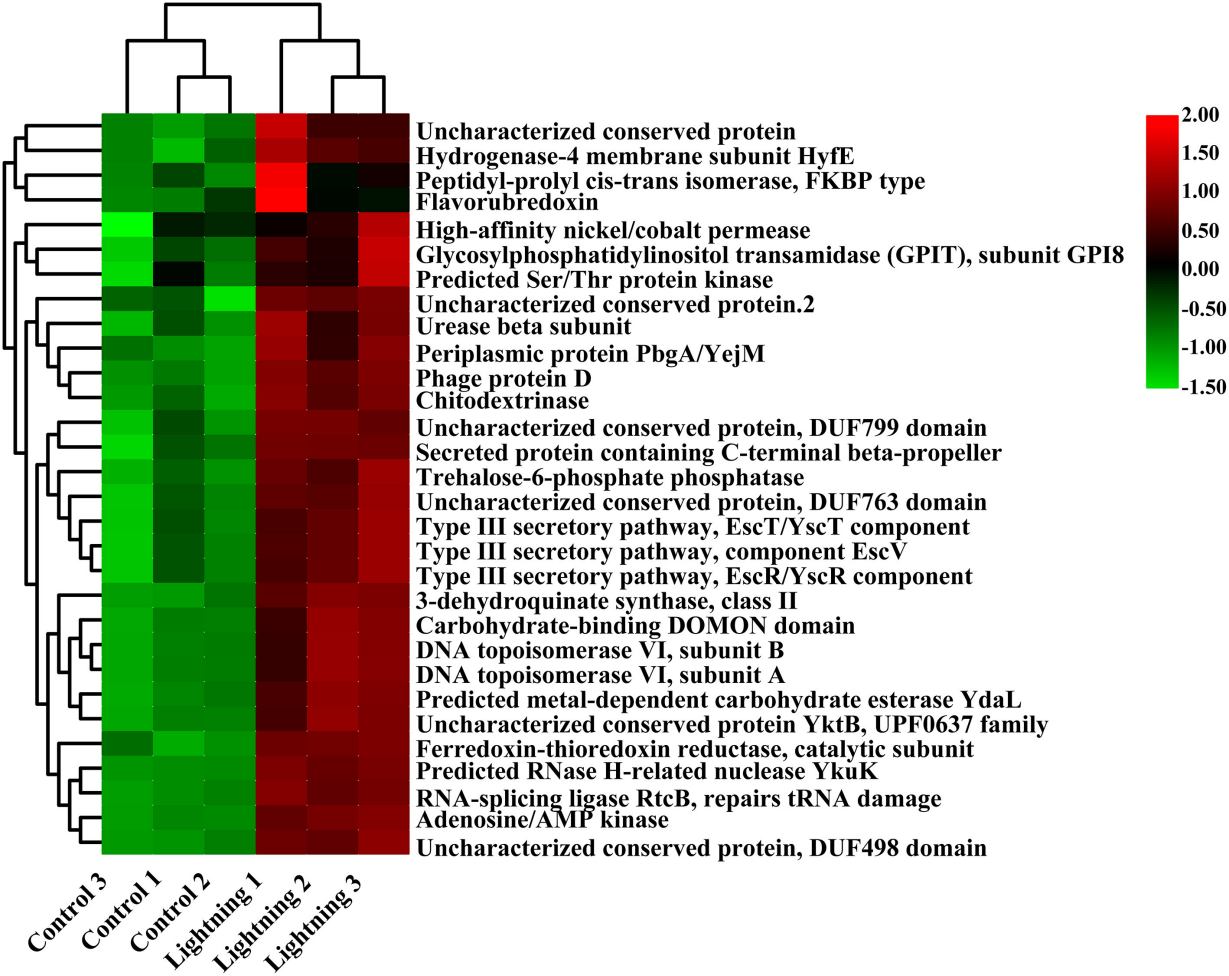
Clusters of Orthologous Groups (COG) functions significantly enriched between different samples (*P* < 0.05). The color block in the heat map from green to red indicates that the abundance of the sample increases gradually. Lighting represents a sample 10 m from the lightning rod; Control indicates that the distance between the sample and the lightning rod is >1,500 m.

**Figure 6:**
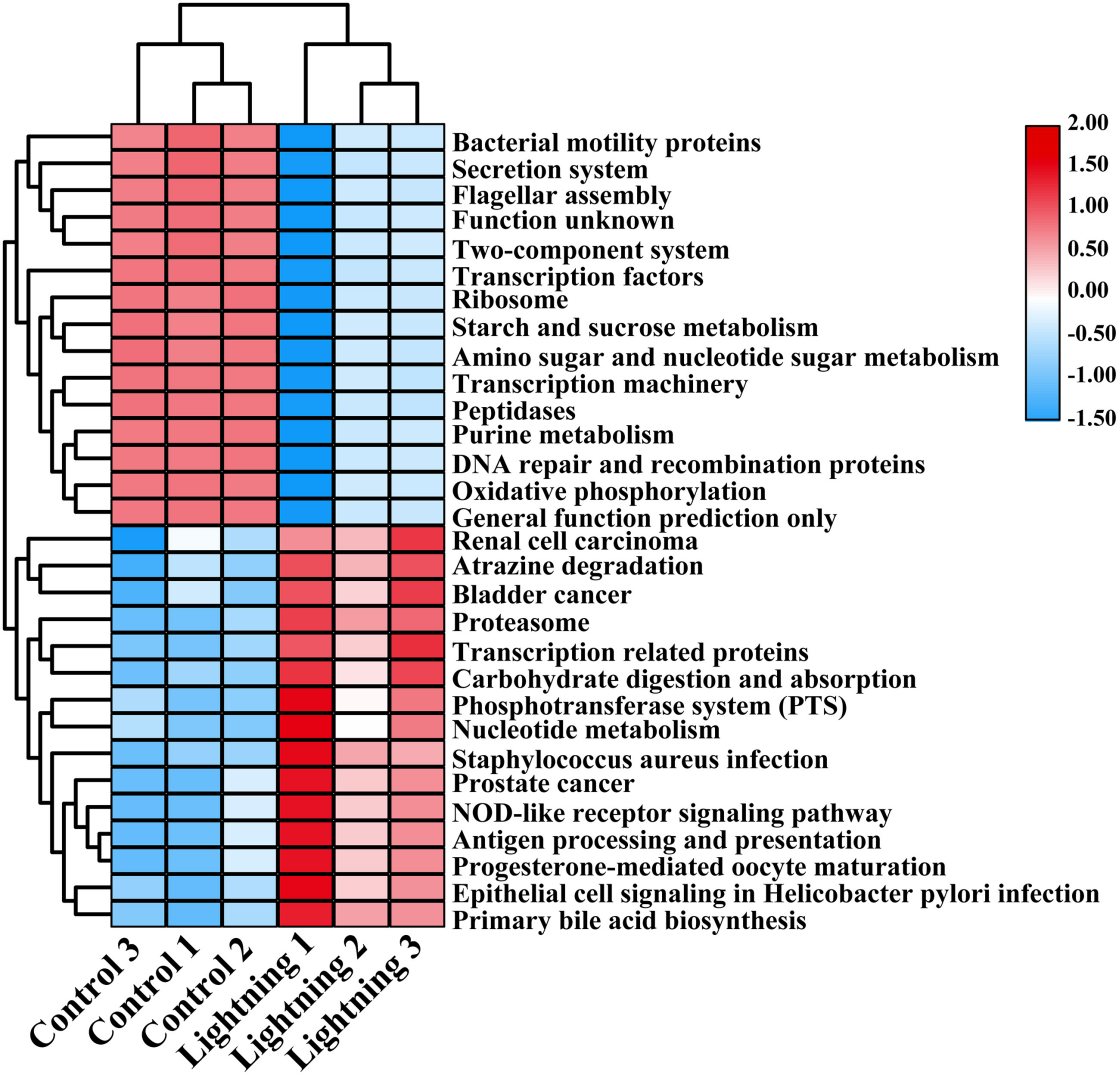
Kyoto Encyclopedia of Genes and Genomes (KEGG) functions significantly enriched between different samples (*P* < 0.05). The color block in the heat map from green to red indicates that the abundance of the sample increases gradually. Lighting represents a sample 10 m from the lightning rod; Control indicates that the distance between the sample and the lightning rod is >1,500 m.

We further predicted the function of bacteria in different rhizosphere soil samples using PICRUSt. The results showed that most of the 30 COG functions with the highest abundance increased in the rhizosphere soil samples near the lightning rod (Figure 5), including adenosine/AMP kinase, chitodextrinase, urease beta subunit, high-affinity nickel/cobalt permease, and flavorubredoxin (*P* < 0.05). KEGG analysis indicated that oxidative phosphorylation, DNA repair and recombination proteins, amino sugar and nucleotide sugar metabolism, starch and sucrose metabolism, the secretion system, and bacterial motility protein pathways were significantly enriched in the CK sample (Figure 6). In addition, primary bile acid biosynthesis, antigen processing and presentation, nucleotide metabolism, carbohydrate digestion and absorption, transcription-related proteins, and proteasome KEGG pathways were significantly enriched in the rhizosphere soil samples near the lightning rod compared with the control samples (*P* < 0.05).

### Bacterial Interaction and Community Structure Differentiation

**Figure 7:**
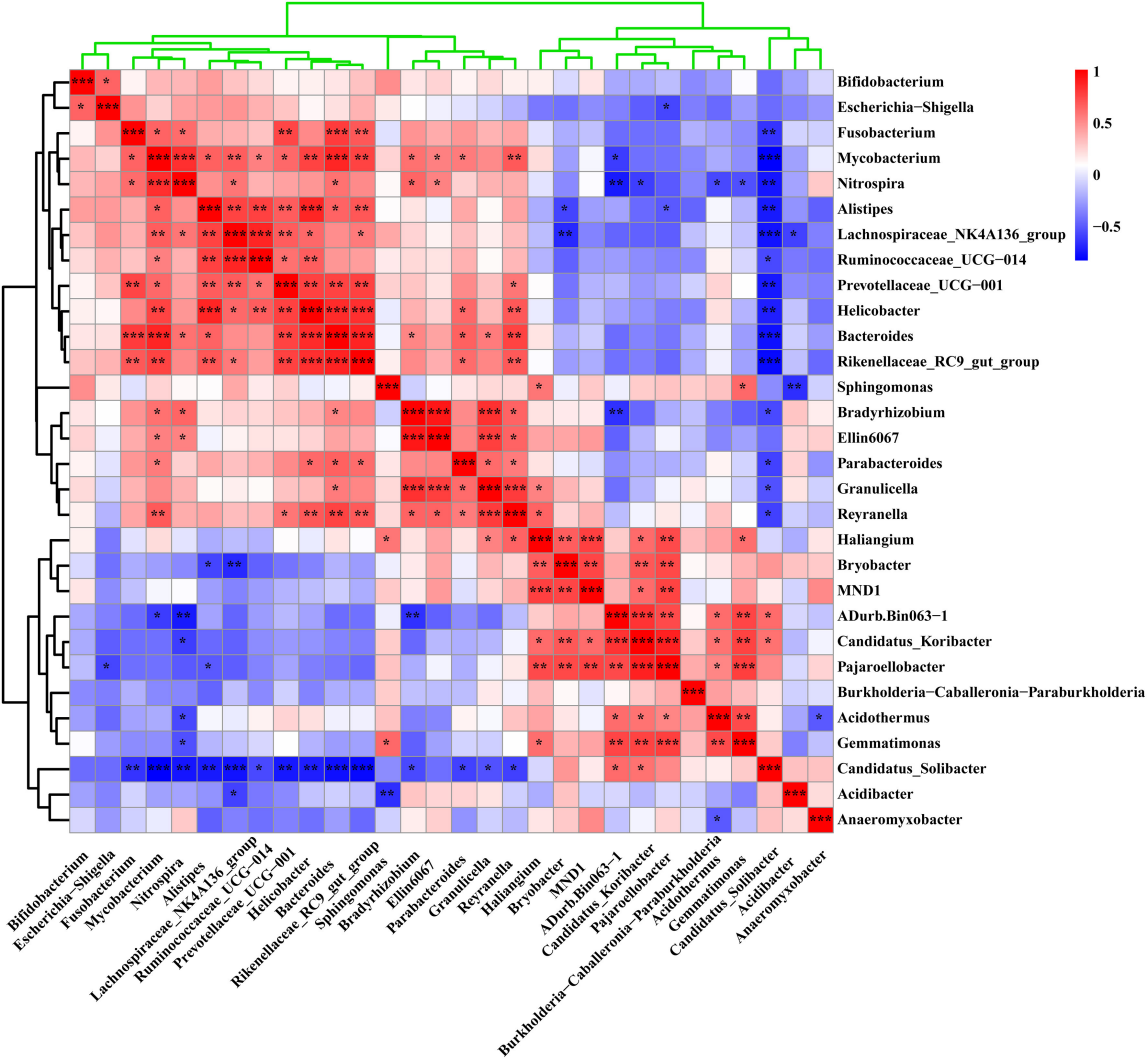
Correlation analysis of the 30 genera with the highest relative abundance in the sample. The color bar in the upper right corner represents the Pearson correlation index of different samples. The blue color block indicates that there is a negative correlation between different samples, while the red color block indicates that there is a positive correlation between different samples. * Indicates a significant correlation between samples at the level of *P* < 0.05, **Indicates a significant correlation between samples at the level of 0.01 < *P* < 0.01, while *** indicates a significant correlation between samples at the level of *P* < 0.001.

We further analyzed the correlation between the rhizosphere soil bacterial communities to analyze their potential interactions based on Pearson correlation index (Figure 7). The results showed a positive or negative correlation between some rhizosphere bacteria. *Sphingomonas, Nitrospira, Reyranella*, and *Mycobacterium*, which were dominant in the soil samples near the lightning rod, had significant correlations with *Gemmatimonas, Bradyrhizobium, Haliangium*, and *Parabacteroides*, respectively (*P* < 0.05). In addition, *Sphingomonas* has a negative correlation with *Acidibacter* (*P* < 0.05). *Nitrospira* and *Reyranella* had a negative correlation with Candidatus_Solibacter (*P* < 0.05). *Mycobacterium* had a negative correlation with ADurb.Bin063–1 (*P* < 0.05). The dominant bacteria in the CK sample, including MND1 and *Candidatus_Solibacter*, showed positive correlations with *Pajaroellobacter* and negative correlations with *Nitrospira* (*P* < 0.05).

**Figure 8:**
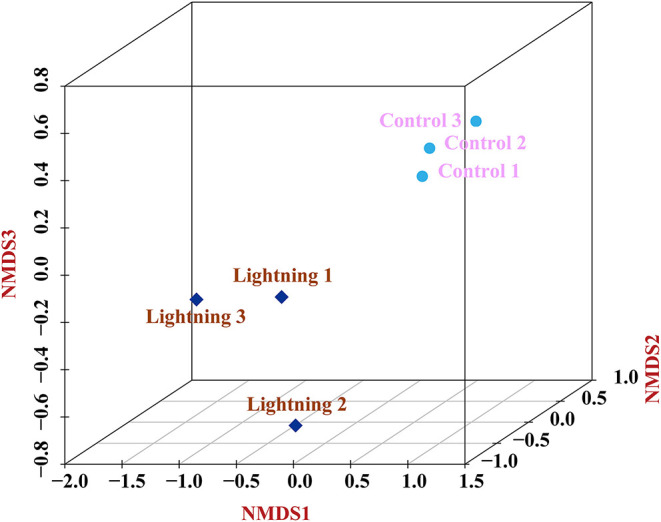
Variations of bacterial communities among different samples based on Nonmetric Multidimensional Scaling (NMDS) analysis. Lighting represents a sample 10 m from the lightning rod; Control indicates that the distance between the sample and the lightning rod is >1,500 m.

We assessed the variations in the bacterial communities between the rhizosphere samples near or far from the lightning rod (Figure 8). The results showed that the bacterial community structure of rhizosphere soils was different from that of the CK sample. The grounding of the lightning rod contributed to the microbial community differentiation.

### Effects of Lightning on Pu-erh Tea Components

**Figure 9:**
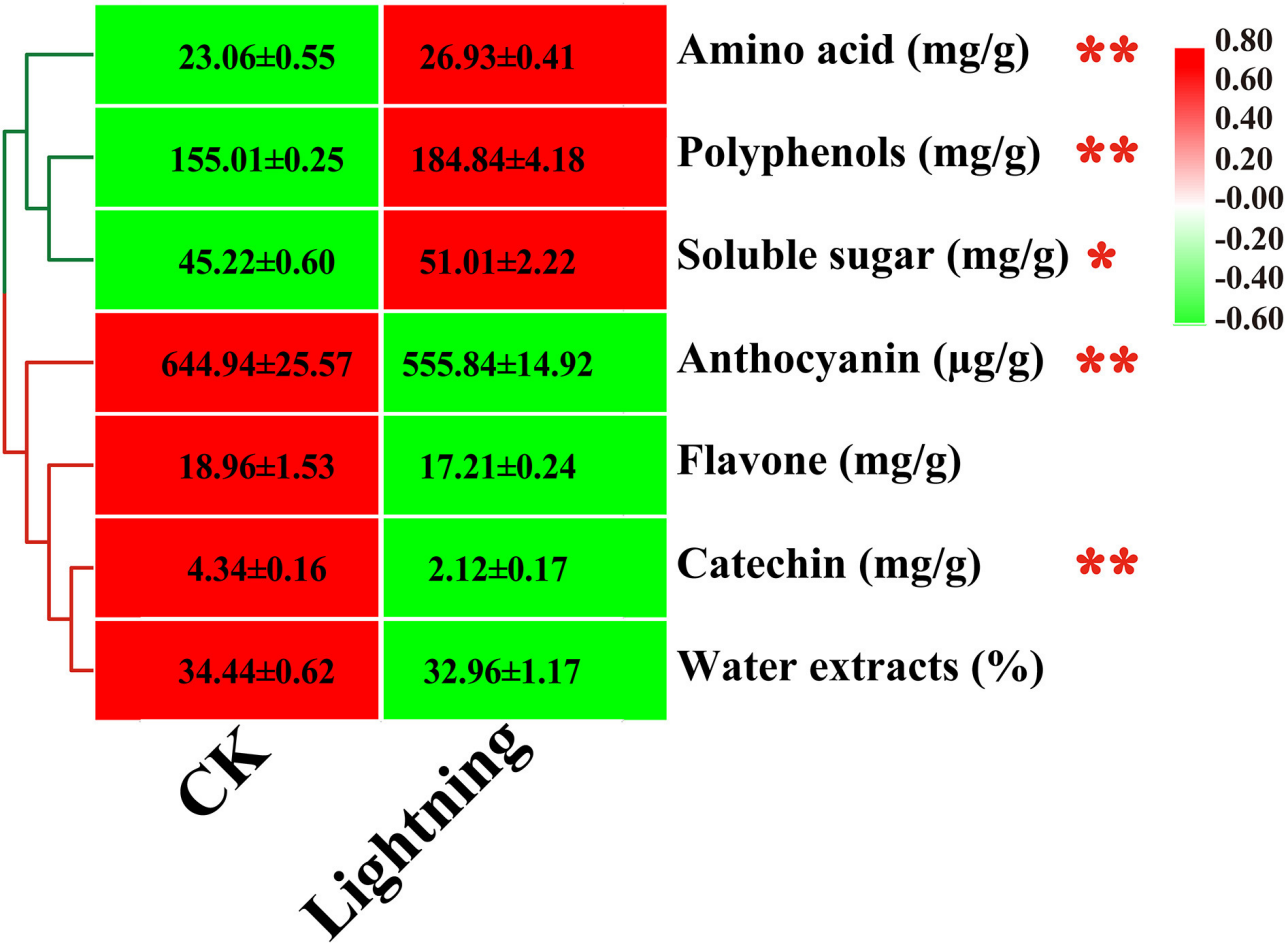
Content of active substances in Pu-erh leaves. The contents of different elements are expressed as mean values ± standard deviation (SD) (mg/kg). Lighting represents a sample 10 m from the lightning rod; CK indicates that the distance between the sample and the lightning rod is >500 m. *Indicates significant differences between different samples at the level of *P* < 0.05, while ** indicates significant differences between different samples at the level of *P* < 0.01.

We further tested the effect of lightning on the active components of Pu-erh tea (Figure 9). The results showed that compared with the control, three active components of Pu-erh tea increased to varying degrees, while four active components decreased to varying degrees. Statistical analysis showed that the contents of amino acids, polyphenols, and soluble sugar increased in Pu-erh tea near the lightning rod, while the contents of catechin and anthocyanin decreased in Pu-erh tea near the lightning rod compared with the control sample (*P* < 0.05).

### Interactions Among Microbial Indicators, Soil Properties, and Pu-erh Tea Components

**Figure 10:**
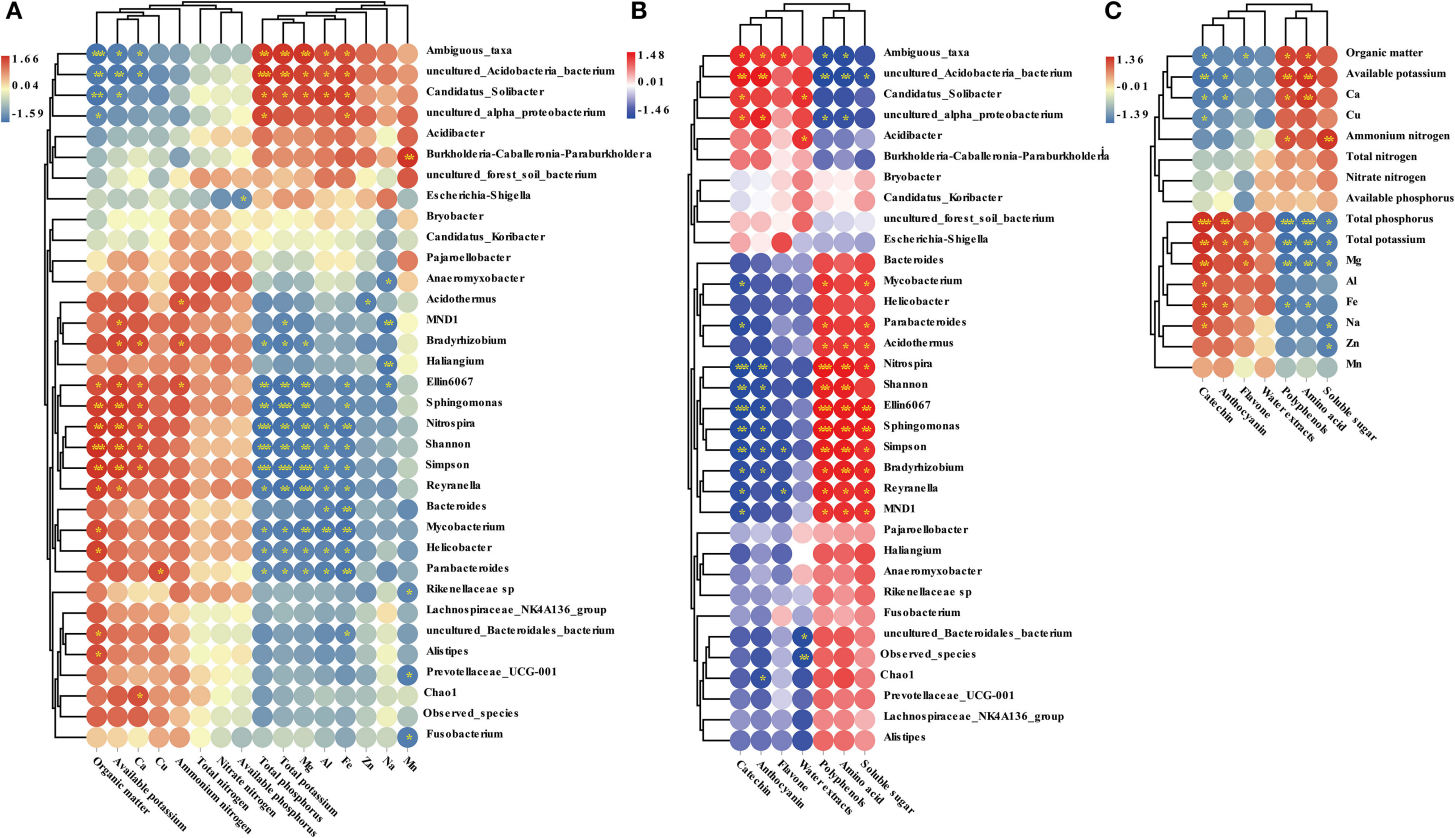
Correlation analysis between soil properties and microbial indicators **(A)**, between Pu-erh active ingredient content and microbial indicators **(B)**, between soil properties and Pu-erh active ingredient content **(C)**. The blue color block indicates that there is a negative correlation between different samples, while the red color block indicates that there is a positive correlation between different samples. *Indicates a significant correlation between samples at the level of *P* < 0.05, **Indicates a significant correlation between samples at the level of 0.01 < *P* < 0.01, while *** indicates a significant correlation between samples at the level of *P* < 0.001.

The Pearson correlation index among microbial indicators, soil properties, and Pu-erh tea components was calculated in this study (Figure 10). We found that the contents of organic matter and available potassium were positively correlated with *Sphingomonas, Nitrospira*, and *Reyranella*. However, the contents of total potassium, total phosphorus, and magnesium were negatively correlated with *Sphingomonas, Nitrospira*, and *Reyranella*. The abundance of *Sphingomonas, Nitrospira*, and *Bradyrhizobium* was positively correlated with amino acids, polyphenols, and soluble sugar and negatively correlated with catechins and anthocyanins. In addition, the contents of available potassium and calcium were positively correlated with amino acids and polyphenols and negatively correlated with catechins and anthocyanins. Total potassium, total phosphorus, and magnesium were negatively correlated with amino acids, polyphenols, and soluble sugar. Total potassium and total phosphorus had a positive correlation with catechins and anthocyanins.

## Discussion

It is estimated that an average of 8 million lightning flashes occur on earth every day. Lightning activities are generally considered harmful to human society and the environment (Ritenour et al., 2008; Middey and Chaudhuri, 2013). Thousands of animals and humans are killed or injured by lightning strikes every year (Browne and Gaasch, 1992; Cankaya et al., 2002). In some areas, lightning strikes have become a major local health risk (Uman, 1994; Rakov and Uman, 2003). In addition, lightning strikes are closely related to forest wildfire, which is one of the main causes of forest ecosystem imbalance and tree death (Clarke et al., 2019; Fill et al., 2019; Gora et al., 2021). Furthermore, an increase in surface pollutants is considered to be closely related to the frequency of lightning (Gharaylou et al., 2020). The use of lightning rods effectively reduces the damage caused by lightning to buildings and the environment. However, the biological effects of lightning rods are unknown.

In this study, we chose Xishuangbanna as the test area for lightning treatment because the average number of lightning strikes exceeds 110 each year. Three large lightning rods were installed in a Pu-erh tea garden. Lightning enters the soil near the lightning rod through the guidance of the lightning rod and affects the physical and chemical properties of the soil and the growth of plants to a certain extent. Statistical analysis showed that the contents of organic matter, available potassium, copper, and calcium in the rhizosphere soil near the lightning rod were significantly higher than those in the control soil (*P* < 0.05), while the contents of total potassium, total phosphorus, iron, magnesium, and aluminum decreased. We found an interesting phenomenon that the total phosphorus content in the soil near the lightning rod decreased and the available phosphorus content increased, indicating that lightning can increase the availability of soil phosphorus. The reasons for the changes in the soil organic matter and element contents need to be further examined.

Lightning also significantly increased the bacterial observed species index and the Shannon and Simpson indices of the Pu-erh rhizosphere soil compared with control soil samples (*P* < 0.05). The diversity of soil microorganisms is closely related to plant growth, stress tolerance, and health (Ishaq, 2017; Bziuk et al., 2021; Fadiji et al., 2021). An increase in soil bacterial diversity may help Pu-erh tea to better cope with a changing environment, diseases, and pests and may increase biomass or quality (Wang et al., 2021; Yang et al., 2021). In addition, *Sphingomonas, Nitrospira*, and *Reyranella* were significantly enriched in soil samples near the lightning rod relative to soil samples far from the lightning rod. *Sphingomonas* has been widely reported to play an important role in ecological restoration and plant growth promotion (Wang et al., 2021). As an aerobic chemolithoautotrophic nitrite-oxidizing bacterium, *Nitrospira* plays an important role in nitrification (Daims and Wagner, 2018). *Reyranella* was successfully isolated from fresh water, rhizosphere soil and plant litter, indicating its wide adaptability (Lee and Whang, 2014; Cui et al., 2017; Lee et al., 2017). The enrichment of these plant-growth-promoting bacteria in the rhizosphere of Pu-erh tea near the lightning rod will have a beneficial effect on the growth of Pu-erh tea. Further analysis showed that lightning led to the differentiation of the bacterial community structure and functional differences in the rhizosphere of Pu-erh tea. Adenosine/AMP kinase, chitodextrinase, flavorubredoxin, nucleotide metabolism, and carbohydrate digestion and absorption were significantly enriched in the rhizosphere soil samples near the lightning rod compared with the control samples (*P* < 0.05). The results showed that microorganisms have different metabolic mechanisms to adapt to the ecological impact of lightning. Lightning has been reported to mediate horizontal gene transfer of soil bacteria (Demaneche et al., 2001; Kotnik, 2013), which is also considered to affect the functional differentiation of soil bacteria.

The contents of amino acids, polyphenols, and soluble sugar were increased in Pu-erh tea near the lightning rod, while the contents of catechins and anthocyanins decreased in Pu-erh tea near the lightning rod compared with the control sample (*P* < 0.05). Pu-erh tea is an important Chinese tea with health value owing to the active substances contained in the leaves (Bond and Derbyshire, 2019; Meng et al., 2019; Zhu et al., 2020). The change in the active substance content in Pu-erh tea shows that lightning had a direct or indirect impact on the metabolism of Pu-erh tea, possibly through the change in soil properties and the rhizosphere microbial structure. We found significant correlations among microbial indicators, soil properties, and Pu-erh tea components. The results verified the interactions among lightning, soil properties, microbial structure, and Pu-erh tea (Liao et al., 2021). This study indicates there may be some biological effect of lightning rods, which we previously ignored. This study serves as the first report on the effects of lightning on soil properties, microecology, and plant growth and promotes the understanding of the biological effects of lightning, providing a reference for the potential use of lightning resources.

## Data Availability Statement

The data presented in the study are deposited in the NCBI repository, accession number SRP372993.

## Author Contributions

YC, QL, and BW: design experiment. WW, XL, XD, and XC: experimental operation. JX, SZ, and WY: data analysis. BW: project management. YC, QL, and JC: manuscript writing and review. All authors contributed to the article and approved the submitted version.

## Funding

This study was financially supported by the Yunnan Science and Technology Major Project (Grant No. 202002AE09001004).

## Conflict of Interest

The authors declare that the research was conducted in the absence of any commercial or financial relationships that could be construed as a potential conflict of interest.

## Publisher's Note

All claims expressed in this article are solely those of the authors and do not necessarily represent those of their affiliated organizations, or those of the publisher, the editors and the reviewers. Any product that may be evaluated in this article, or claim that may be made by its manufacturer, is not guaranteed or endorsed by the publisher.

## Supplementary Material

Supplementary Figure S1 Rarefaction curves of bacterial OTUs in different samples. Lighting represents a sample 10 m from the lightning rod; Control indicates that the distance between the sample and the lightning rod is >1,500 m.
