# Supplementary figures and images for: Effects of Lightning on Rhizosphere Soil Properties, Bacterial Communities, and Active Components of Camellia sinensis var. assamica
Source: Front Microbiol. 2022 May 23;13:911226. doi: 10.3389/fmicb.2022.911226 (PMC9169052; doi:10.3389/fmicb.2022.911226)

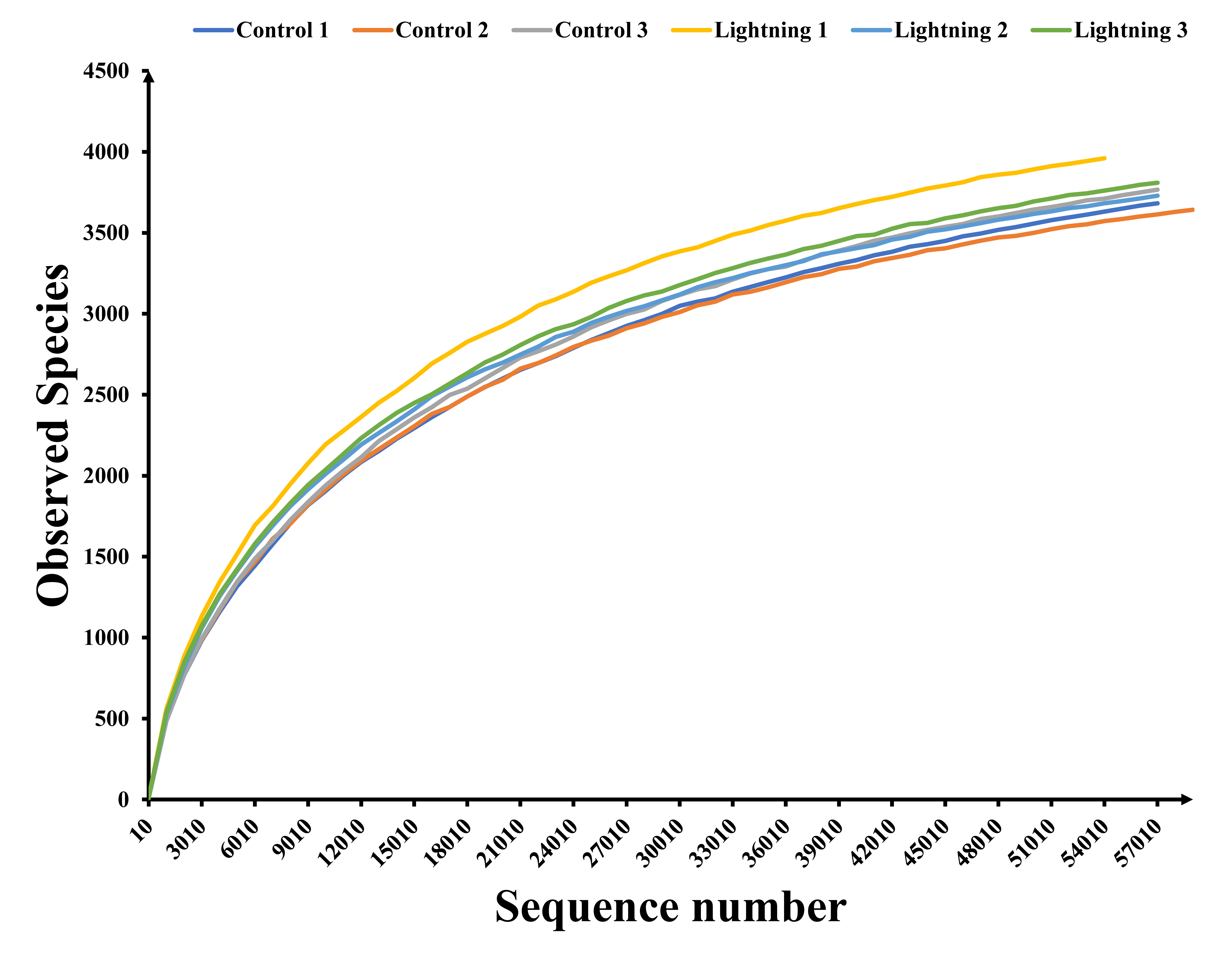

Supplement: Supplementary Figure S1 — Rarefaction curves of bacterial OTUs in different samples. Lighting represents a sample 10 m from the lightning rod; Control indicates that the distance between the sample and the lightning rod is >1,500 m. [file Image_1.TIF]
